# The *in vivo* performance of small-caliber nanofibrous polyurethane vascular grafts

**DOI:** 10.1186/1471-2261-12-115

**Published:** 2012-12-03

**Authors:** Zuo-jun Hu, Zi-lun Li, Ling-yu Hu, Wei He, Rui-ming Liu, Yuan-sen Qin, Shen-ming Wang

**Affiliations:** 1 Department of Vascular Surgery, The First Affiliated Hospital of Sun Yat-Sen University, No. 58 Zhongshan 2nd Road, Guangzhou, 510080, China; 2 Guangzhou Women and Children’s Medical Center, Guangzhou, 510080, China

**Keywords:** Nanofibers, Polyurethane, Small-caliber vascular grafts, Animal study

## Abstract

**Background:**

In a previous *in vitro* study, we confirmed that small-caliber nanofibrous polyurethane (PU) vascular grafts have favorable mechanical properties and biocompatibility. In the present study, we examined the *in vivo* biocompatibility and stability of these grafts.

**Methods:**

Forty-eight adult male beagle dogs were randomly divided into two groups receiving, respectively, polyurethane (PU) or polytetrafluoroethylene (PTFE) grafts (n = 24 animals / group). Each group was studied at 4, 8, 12, and 24 weeks after graft implantation. Blood flow was analyzed by color Doppler ultrasound and computed tomography angiography. Patency rates were judged by animal survival rates. Coverage with endothelial and smooth muscle cells was characterized by hematoxylin-eosin and immunohistological staining, and scanning electron microscopy (SEM).

**Results:**

Patency rates were significantly higher in the PU group (p = 0.02 vs. PTFE group). During the first 8 weeks, endothelial cells gradually formed a continuous layer on the internal surface of PU grafts, whereas coverage of PTFE graft by endothelial cells was inhomogeneous. After 12 weeks, neointimal thickness remained constant in the PU group, while PTFE group showed neointimal hyperplasia. At 24 weeks, some anastomotic sites of PTFE grafts became stenotic (p = 0.013 vs. PU group). Immunohistological staining revealed a continuous coverage by endothelial cells and an orderly arrangement of smooth muscle cells on PU grafts. Further, SEM showed smooth internal surfaces in PU grafts without thrombus or obvious neointimal hyperplasia.

**Conclusions:**

Small-caliber nanofibrous PU vascular grafts facilitate the endothelialization process, prevent excessive neointimal hyperplasia, and improve patency rates.

## Background

Diseases resulting from vascular atherosclerosis are among the leading causes of death [1,2]. Vascular grafting is a vital treatment option for severe diseases caused by atherosclerosis, such as cardiovascular diseases. Currently, large-caliber vascular grafts made of polytetrafluoroethylene (PTFE) or polyethylene terephthalate (PET) have been successfully used in the clinic [3-6]. However, synthetic vascular grafts with calibers of <6 mm are associated with relatively low long-term potencies and generally produce unsatisfactory results. Therefore, small-caliber synthetic vascular grafts are more desirable for replacement of blood vessels blocked by severe stenosis or occlusion, such as done during cardiovascular bypass grafting or peripheral vascular bypass grafting.

Synthetic vascular grafts have been studied for nearly five decades. The principal candidate materials identified as suitable for these grafts include PET, expanded PTFE, silk, and polyurethane (PU). Among these, PU has attracted the most interest because of its good antithrombogenicity and excellent compliance, beneficial physical / mechanical properties, and biocompatibility. Furthermore, the surface of PU can be biomimetically modified to even further improve its blood compatibility and, thus, increase the long-term graft patency. Indeed, satisfactory blood compatibility is one of the most desirable features of a vascular graft.

In a previous study [7], we prepared nanofibrous, small-caliber PU vascular grafts by electrospinning. Those grafts had the following advantages: favorable fiber orientation, excellent tensile properties, and an *in vitro* biocompatibility with human endothelial cells [8]. In the present study, we further tested the *in vivo* biocompatibility, blood compatibility, and stability of these electrospun PU grafts. Our objective was to evaluate the potential of these grafts for clinical applications.

## Methods

### Vascular grafts

**Figure 1:**
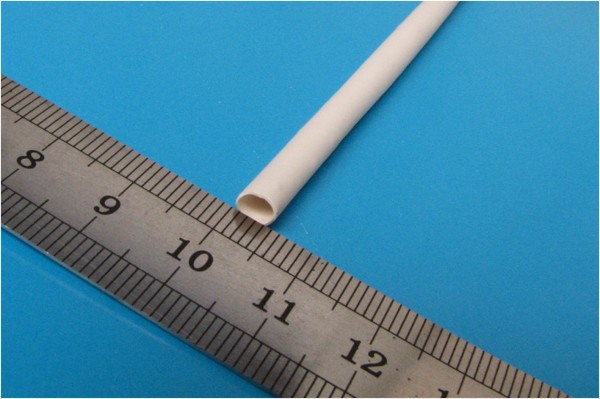
**Electrospun small**-**caliber nanofibrous PU vascular graft(inner diameter of 4 mm).**

Nanofibrous PU vascular grafts with an inner diameter of 4 mm (Figure 1) were prepared in our laboratory by electrospinning with MDI-polyester/polyether polyurethane (CAS number: 68084-39-9; Sigma-Aldrich). PTFE vascular grafts (inner diameter of 4 mm) were purchased from WL Gore & Associates (Newark, USA).

### Animals

The use of animals in this study was approved by the Animal Ethics Committee of our Hospital. Forty-eight adult male beagle dogs (9 – 10 month old, 10 – 12 kg weight) were purchased from Guangzhou Institute of Pharmaceutical Industry (Guangzhou, Guangdong, China). Each dog was caged separately and maintained in the Center of Experimental Animals of Sun Yat-sun University. The dogs were fed with standard food, and the cages were regularly disinfected by UV irradiation.

The dogs were randomly divided into two groups, 24 animals each, and implanted either PU or PTFE grafts. Then, the dogs in each group were assigned to four experimental time points (4, 8, 12, and 24 weeks), with 6 dogs per each time point.

### Surgical procedures

Before surgery, the dogs fasted for 24 hours and ceased drinking for 6 hours. The dogs were anaesthetized by intramuscular injection of 0.1 mg/kg of Sumianxin (Institute of Veterinary Medicine, Military Supplies University, Changchun, Jilin, China), followed by intraperitoneal injection of 30 ml/kg of 3% pentobarbital (Merck, Darmstadt, Germany). The anesthesia was monitored and, when necessary, maintained by infusions of 1 – 2 ml of 3% pentobarbital through the ear vein. An 8-gauge endotracheal tube was inserted through a dog’ mouth, and vital parameters (e.g., breath rate, heartbeat, body temperature) were closely monitored. Cefradine (1 g) was intravenously infused 30 min before surgery.

**Figure 2:**
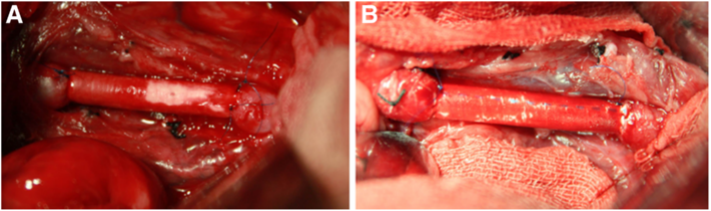
**Anastomoses of both ends.** (**A**) A PU graft, (**B**) A PTFE graft.

Restrained dogs were placed in supine position on the operation table and continuously infused with 5% glucose / saline solution until completion of surgery. The abdominal area was shaved and disinfected with Anduofu disinfectant (ADF, Shenzhen, Guangdong, China). A 20-cm incision was made along the abdominal midline, the intestines were pulled out and covered with wet gauze, and the spleen was repositioned in the upper left abdomen. The retroperitoneum was incised to expose the abdominal aorta. Second lumbar arteries were severed with care to keep the inferior mesenteric artery intact. In some cases, third lumbar arteries were also severed. After separating and exposing the abdominal aorta, 10 ml of heparinized saline solution (125 U/ml) were intravenously injected. The abdominal aorta was clamped at the proximal and the distal ends (i.e., slightly below the inferior mesenteric artery, or above the iliac artery bifurcation, or at the level of third lumbar arteries) with aortic clamps to stop the blood flow. An approximately 2-cm long segment of the abdominal aorta was resected. A vascular graft was selected and anastomosed to the proximal end and then to the distal end of the artery, with both ends sutured with 6–0 Prolene sutures (Johnson & Johnson, New Jersey, USA). The proximal clamp was then released to allow blood to fill the graft, and expel air and blood clots. During this, the 5% glucose-saline infusion rate was increased. Then, the distal clamp was gradually released to restore the blood flow (Figure 2). Finally, the wounds were closed in a standard fashion.

### Post-surgical care

The dogs were returned to their cages and maintained at 25°C. The next day after surgery, the dogs were treated with analgesia and allowed free access to water and food. They also received intramuscular injection of cefradine (0.5 g/injection, twice daily) to prevent infection.

The dogs were not treated with any anticoagulant drugs. Movements of the hind limbs were daily examined for the signs of spinal cord ischemia. The graft patency was estimated by computed tomography angiography (CTA; Toshiba Aquilion 64, Tokyo, Japan), as well as by manual palpation of the femoral artery pulsation.

### Sample harvest

In each group, six samples were harvested at each time point (i.e., 4, 8, 12, and 24 weeks) after the surgery. Under general anesthesia, the grafts were retrieved along with the host blood vessels 1 cm above from the graft, labeled for anatomic orientation, and gently rinsed with PBS to remove residual blood. Anastomotic sites were photographed. The grafts were longitudinally dissected, and the patency and tissue formation on the inner surface were observed and photographed. Then, the grafts were transversely cut into two parts. One part was used for the subsequent histological examinations, while the other for scanning electron microscopy observation. After harvesting, the dogs were euthanized by pentobarbital overdose.

### Histological examinations

The samples were observed for patency, compliance, formation of aneurysm and aortic dissection, stenosis at the anastomotic sites, and the interaction between the external wall and the adjacent host tissues. The grafts were longitudinally dissected to check the smoothness of the anastomotic sites and the presence of mural thrombus and neointimal hyperplasia.

The samples were fixed in 10% neutral formaldehyde for 12–24 hours. Three smaller specimens were collected from the proximal end, medium part, and distal end of the specimen. The samples were embedded in paraffin and serially sectioned into 10 slides, each 2 μm thick. Slides were stained with hematoxylin / eosin and examined under microscope (Nikon Eclipse 80i; Nikon, Tokyo, Japan) to estimate neointimal thickness.

The slides were stained for von Willebrand factor (vWF) and smooth muscle actin using UltraSensitive SP kits (KeyGenTec, Nanjing, Jiangsu, China). The primary antibodies were polyclonal rabbit IgG against human vWF-associated antigen and monoclonal rat IgG against human smooth muscle actin (both from Santa Cruz Biotechnology, California, USA). The secondary antibodies were biotin-labelled goat anti-rabbit IgG and goat anti-mouse IgG (Cytodiagnostics, Burlington, Ontario, Canada). Slides were stained with primary antibody according to the manufacturer’s instructions, developed with DAB, and counterstained with hematoxylin. Harvested host vessels served as positive controls.

Slides were examined under microscope (Nikon Eclipse 80i) and images were captured using the Nikon ACT-2U graphics program supplied with the microscope. Endothelial cells in the intimal surface bearing brown particles in the cytoplasm were classified as positive for Factor VIII. Smooth muscle cells inside the intima bearing brown particles in the cytoplasm were identified as positive for α-actin. The coverage of vascular endothelial cells and smooth muscle cells on the internal surface of the graft was evaluated in a similar fashion.

### Scanning electron microscopy

The samples were fixed in 2.5% glutaraldehyde for 4 hours, immersed in PBS overnight, followed by six rinses with PBS. Samples were then dehydrated in series of ethanol solutions of ascending concentrations (30%, 50%, 70%, and 90%; 2 × 15 min each), absolute ethanol (2 × 15 min), tert-butanol (3 × 15 min), and freeze dried for 3 hours. Dried samples were sputter-coated with gold and studied with an FE Quanta 200 scanning electron microscope (SEM). Samples were observed at low magnifications to evaluate the growth of endothelial cells on the internal surface of the graft, formation of neointima, and the presence of mural thrombus. The morphology and distribution of the endothelial cells were studied at higher magnifications.

### Statistical analysis

Data are shown as mean ± SD and analyzed by unpaired *t* test and one-way ANOVA analysis using the Statistics Software 18 (SPSS, Chicago, USA). Categorical data were analyzed by chi-square test. Differences in patency between two groups were analyzed by survival analysis. The *p* value of < 0.05 was considered statistically significant.

## Results

### General observations

As described above, 48 adult male beagle dogs were randomly divided into two groups which were implanted with either PU or PTFE grafts. The dogs in each group were assigned to four experimental time points (4, 8, 12, and 24 weeks post-surgery), with 6 dogs per each time point. Four weeks after the surgery, one dog with a PU graft died due to rupture at the anastomotic site. The post-mortem anatomical analysis revealed that the graft was patent. Therefore, this dog was included in the analysis at the 4-week time point. Other dogs survived for 8 weeks. The operation time and cross-clamp time were comparable between two groups. Table 1 summarizes general conditions of the animals and surgical procedures.

**Table 1:** General and surgical parameters of study groups

	**PU group**	**PTFE group**
Number of dogs	24	24
Age (months)	9.0 ± 0.3	9.0 ± 0.4
Weight (kg)	10.6 ± 0.4	10.7 ± 0.5
Operation time (min)	143.5 ± 30.1	145.4 ± 30.6
Cross-clamp time (min)	68.6 ± 17.6	71.2 ± 20.5

### Graft patency

**Figure 3:**
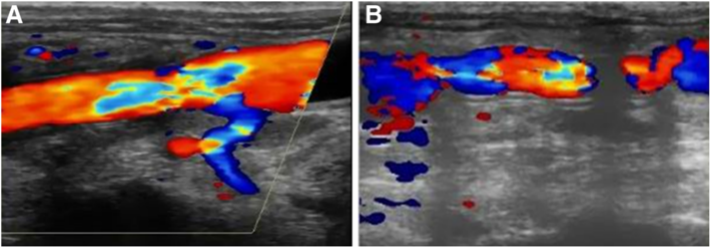
**Color Doppler ultrasound images taken 12 weeks after the surgery.** (**A**) A PU graft, (**B**) A PTFE graft.

**Figure 4:**
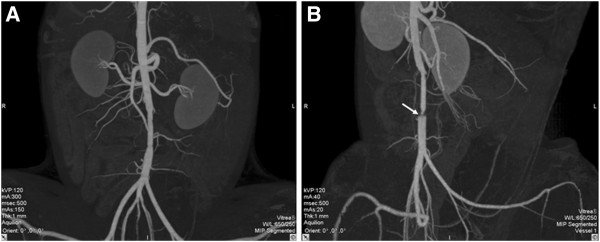
**CT images taken 24 weeks after the surgery(i.e.,shortly before the sample harvest.)** (**A**) A PU graft showing excellent patency, smooth internal wall, and absence of stenosis. (**B**) A PTFE graft showing patency, yet clear stenosis at the distal anastomotic site (marked with white arrow).

**Figure 5:**
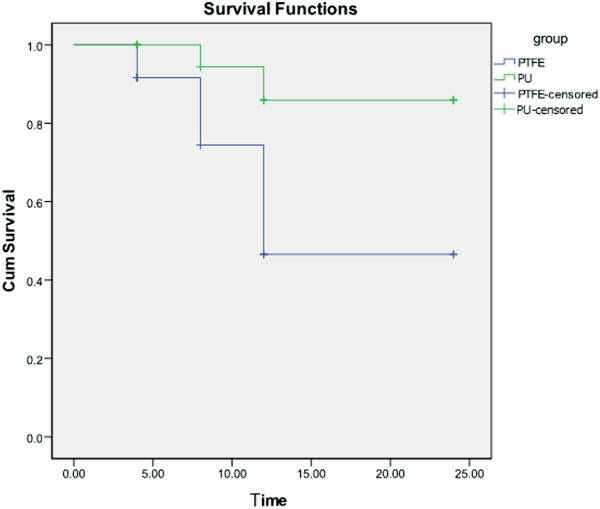
Comparison of patency rates of the PU and PTFE grafts.

Graft patency was verified using a combination of CTA, color Doppler ultrasound, and manual palpation of the femoral artery pulsation (Figures 3 and 4). The dogs were carefully monitored for mental conditions, food intake, hind limb temperature, and physical activity. In the first week after the surgery, the dogs were palpated daily for femoral artery pulsation. After that, the dogs were palpated once a week. Further, experimental animals were examined by color Doppler imaging 2 weeks after the surgery, followed by examinations every 4 weeks. The CTA was done shortly before the sample harvest. The patency rates were quantified using the results from color Doppler and CTA analyses. Table 2 shows the patency rates of two groups at given time point. The survival analysis revealed that the patency rate was significantly higher in the group with PU grafts compared with the group with PTFE grafts (p = 0.02; Figure 5).

**Table 2:** Patency rates of vascular grafts

	**Week 4**	**Week 8**	**Week 12**	**Week 24**
PU group	24 / 24 (100%)	18 / 18 (100%)	11 / 12 (92%)	5 / 6 (83%)
PTFE group	24 / 24 (100%)	16 / 18 (89%)	7 / 12 (58%)	2 / 6 (33%)

Footnote: At any given time point, the patency rate was defined as a ratio between the number of patent grafts and number of dogs alive at that particular time point.

### Histological examinations

No aortic dissection or aneurysm was observed in any graft. Various degrees of collateral circulation were observed around the stenotic or occluded grafts. The PU grafts appeared to be biocompatible, their external surfaces were encapsulated by a small amount of fibrous tissue. By contrast, the PTFE grafts appeared yellowish, moderately tangled, and substantially stiffer. Further, their external surfaces were encapsulated by a much larger amount of fibrous tissue.

The grafts were dissected to reveal tissue formation on their internal surfaces. Four weeks after the surgery, both PU and PTFE grafts were covered by a thin layer of tissue. Eight weeks after the surgery, both grafts were lined by smooth neointima. Twelve weeks after the surgery, the neointima on the PU grafts had a relatively uniform thickness. In contrast, the neointima on the PTFE grafts was not uniform in thickness and showed regional yellow dots. Twenty-four weeks after the surgery, the thickness of the neointima on the PU grafts no longer appeared thickened. In comparison, the PTFE grafts became relatively stiff and the neointima covering them appeared to become thicker with a various extent at different locations.

**Figure 6:**
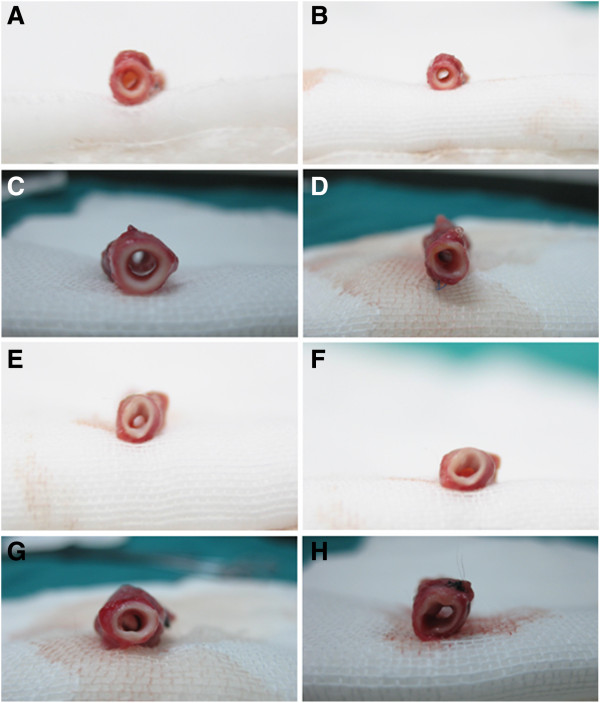
**Anastomotic sites at various time points.** (**A**, **C**, **E**, and **G**) PU grafts showing smoothness and patency at the anastomotic sites without clear stenosis. (**B**, **D**, **F**, and **H**) PTFE grafts with clear stenosis at the anastomotic sites.

**Figure 7:**
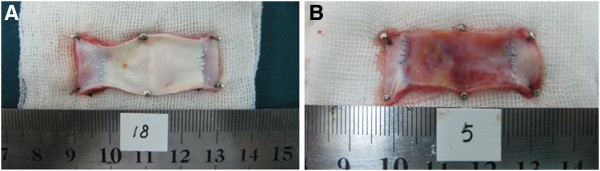
**Internal surfaces of vascular grafts harvested 24 weeks after the surgery.** (**A**) A PU graft showing a smooth and intact surface without thrombus or obvious neointimal hyperplasia. (**B**) A PTFE graft showing uneven surface and obvious neointimal hyperplasia (yellowish and stiffer).

**Figure 8:**
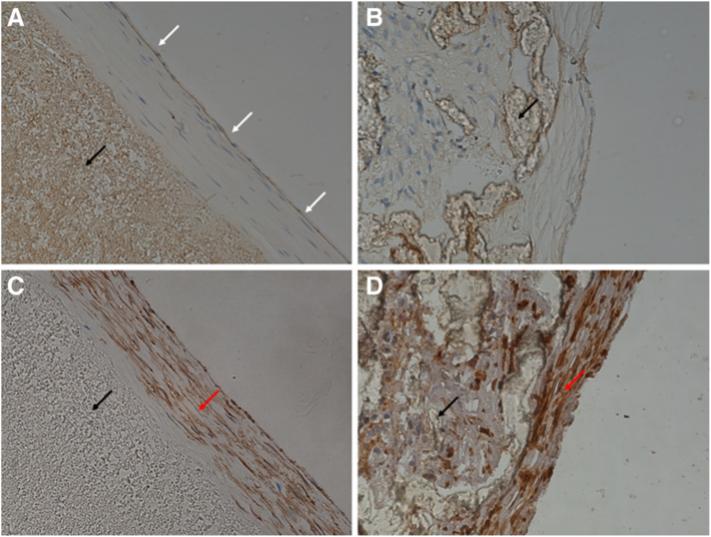
**Neointima on grafts harvested 4 weeks after the surgery(× 400).** White arrows indicate endothelial cells, black arrows vascular grafts, and red arrows smooth muscle cells. (**A**) A PU graft stained for von Willebrand factor (vWF) showing continuous endothelial layer. (**B**) A PTFE graft stained for vWF showing discontinuous endothelial layer. (**C**) A PU graft stained for α-SM-actin showing an orderly arrangement of smooth muscle cells. (**D**) A PTFE graft stained for α-SM-actin showing disordered arrangement of smooth muscle cells.

**Figure 9:**
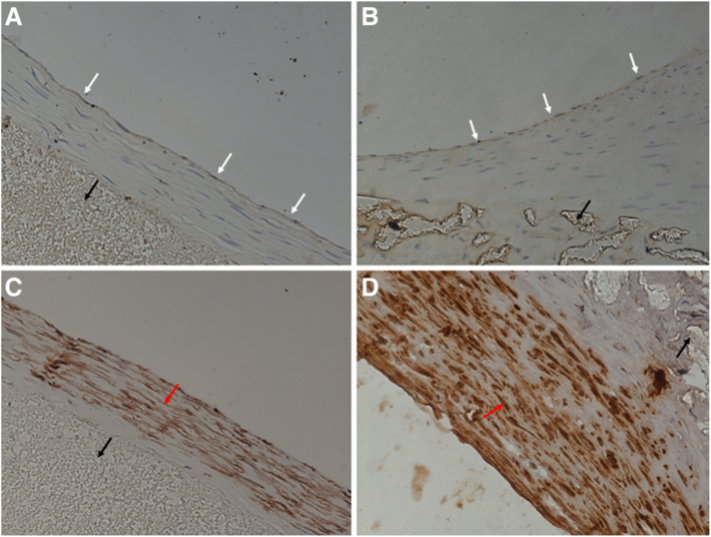
**Neointima on grafts harvested 24 weeks after the surgery(× 400)**. White arrows indicate endothelial cells, black arrows vascular grafts, and red arrows smooth muscle cells. (**A**) A PU graft stained for von Willebrand factor (vWF) showing continuous endothelial layer. (**B**) A PTFE graft stained for vWF showing continuous endothelial layer. (**C**) A PU graft stained for α-SM-actin showing an orderly arrangement of smooth muscle cells without further increase of neointimal thickness. (**D**) A PTFE graft stained for α-SM-actin showing disordered arrangement of smooth muscle cells and increased neointima thickness.

**Figure 10:**
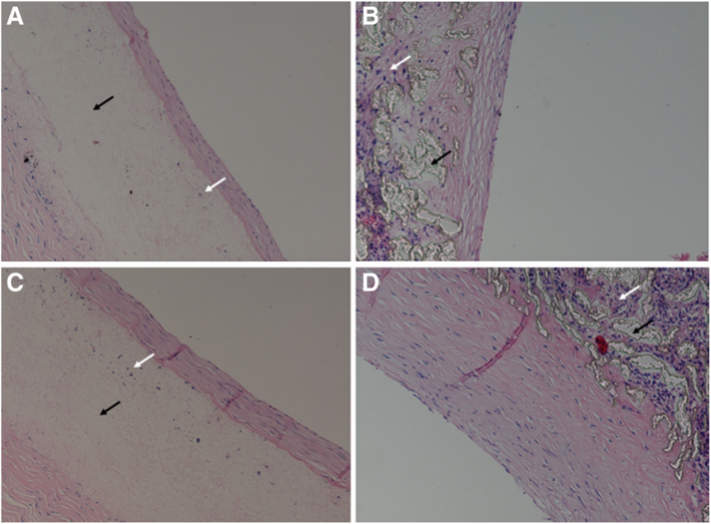
**Interaction of vascular grafts with the host tissue(× 200).** Black arrows indicate vascular grafts, and white arrows indicate in-grown tissue. (**A**) A PU graft harvested 4 weeks after the surgery showing limited ingrowth of fibrous tissue. (**B**) A PTFE graft harvested 4 weeks after the surgery showing more extensive ingrowth of fibrous tissue. (**C**) A PU graft harvested 24 weeks after the surgery still showing limited ingrowth of fibrous tissue and no neointima thickening. (**D**) A PTFE graft harvested 24 weeks after the surgery showing substantially increased tissue infiltration and increased thickness of neointima.

**Figure 11:**
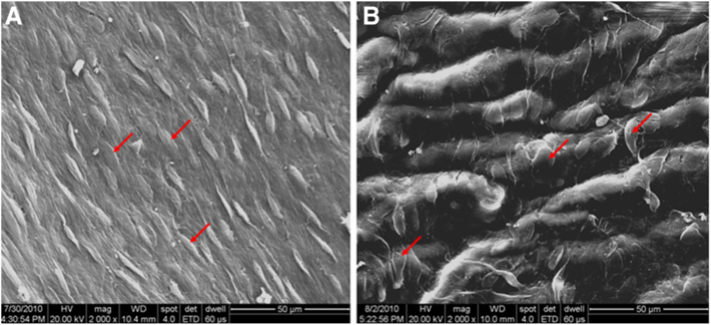
**Endothelial cells on the internal surfaces of grafts harvested 8 weeks after the surgery(× 2000).** Red arrows indicate endothelial cells. (**A**) A PU graft showing complete and continuous endothelial layer lining the inner surface of the graft, well-oriented along the direction of blood flow. (**B**) A PTFE graft showing complete endothelial layer with disordered arrangement and lack of polarity orientation.

**Figure 12:**
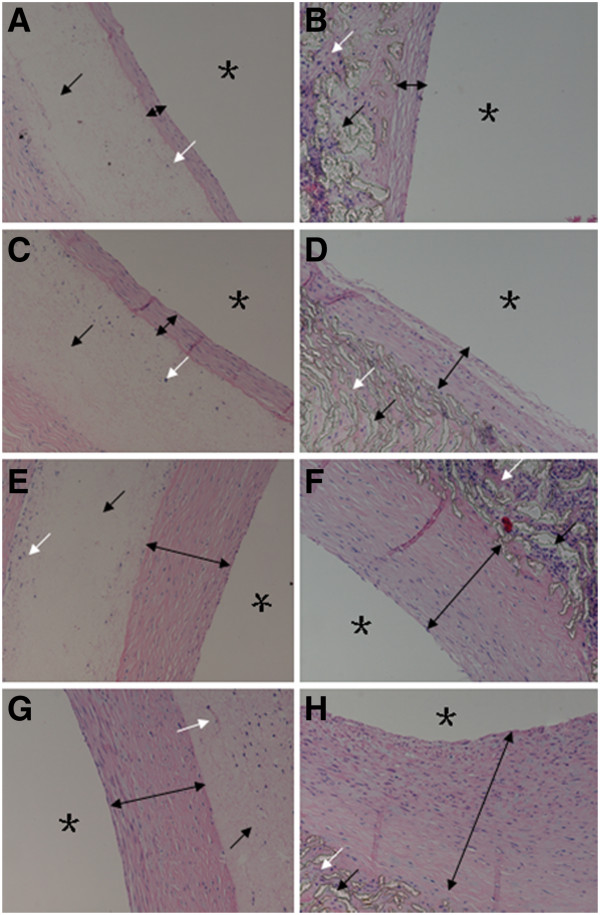
**Neointimal thicknesses at different time points(× 200).** Black arrows indicate vascular grafts, white arrows ingrown tissue, black double arrows neointimal thicknesses, and black stars the graft lumens. (**A**), (**C**), (**E**), and (**G**): A PU graft showing insignificant neointimal hyperplasia. (**B**), (**D**), (**F**), and (**H**): A PTFE graft showing obvious neointimal hyperplasia.

The endothelialization of the grafts was analyzed by the hematoxylin / eosin and immunohistochemistry stainings, and SEM. Overall, the results of these characterizations indicated that endothelialization went faster on the internal surfaces of the PU grafts compared with the PTFE grafts. Four weeks after the surgery, endothelial cells adhered to the PU grafts, yet the cell distribution still remained irregular. However, even fewer endothelial cells adhered to the PTFE grafts at this time point. Eight weeks after the surgery, a continuous endothelial cell layer formed on the PU grafts, suggesting the completion of endothelialization. By contrast, while the PTFE grafts were covered by higher numbers of endothelial cells, the endothelial layer remained heterogenous. Twelve and 24 weeks after the surgery, the endothelialization of both grafts was complete and bilayer (i.e., intima-media) structure formed, similar to host vessels. The PU grafts showed an insignificant neointimal hyperplasia, in contrast to an obvious neointimal hyperplasia inside the PTFE grafts (Figures 6, 7, 8, 9, 10, 11, 12, Table 3).

**Table 3:** Comparison of thicknesses of neointimas between the PU and PTFE vascular grafts

**Time point(weeks)**	**PU group(μm)**	**PTFE group(μm)**	**p**
4	27.3 ± 1.8	24.5 ± 4.8	0.075
8	63.2 ± 3.7	67.4 ± 6.2	0.274
12	71.0 ± 3.5	98.3 ± 13.5	0.103
24	82.2 ± 4.5	170.3 ± 40.5	0.013

## Discussion

Currently, synthetic vascular grafts produced from Dacron or expanded large caliber PTFE are successfully used in the repair of large vessels. However, synthetic grafts with an internal diameter of less than <6 mm are associated with high failure rates because of thrombogenesis or neointimal hyperplasia and the resultant grafting stenosis or occlusion [9-11]. Compared with synthetic vascular grafts, autografts can provide a superior patency rate [12] for two critical reasons: they have the best mechanical / biological match with both recipient site and monolayer of endothelial cells lining their internal surfaces. Endothelial cells not only act as a barrier between the vascular wall and the blood, but also prevent platelet aggregation and thrombus formation and blood coagulation. Rapid endothelialization of synthetic vascular grafts is expected to improve their patency after grafting.

Different synthetic grafts vary in the time required for endothelialization, typically ranging from 10 days to 6 months [13-16]. Endothelialization is affected by multiple factors, including patient’s ethnicity and age, and presence of other disorders. It was found that, within one week after grafting, intima is formed at the anastomotic site by cells migrating from the host vessel; this intima then extends toward the center of the graft [17]. Four weeks after grafting, the graft is encapsulated by the adjacent tissue and fibrous connective tissue, and host endothelial cells penetrate the micropores of the graft and form new capillary vessels providing endothelial cells for formation of the neointima [18,19]. In addition, blood cells may also sludge in the formation of intima on the graft surface. After grafting, the surface of vascular graft is initially covered with a thin layer of thrombi, which then gradually transforms into a fibrous membrane. Endothelial cells colonize this membrane, differentiate, and finally completely cover the internal surface of the graft, completing the endothelialization [20,21].

Three mechanisms have been proposed to explain the process of graft endothelialization. The first mechanism is cell crawling; under this scenario, host vascular endothelial cells and smooth muscle cells crawl across the anastomotic site onto the graft to form the neointima. The second mechanism is cell penetration, when capillary vessels are supposed to penetrate into the micropores of the graft and provide endothelial cells for formation of neointima. Finally, the third mechanism is cell precipitation-diffusion, when cells from blood circulation precipitate on the internal surface of the graft to form the neointima. These mechanisms may be complementary and may all contribute to the endothelialization. However, some studies found that endothelial cells in the adjacent host vessel were not able to migrate for more than 1 cm and thus may not meet the requirement for complete endothelialization of the graft. Additionally, pores of excessively large sizes may permit exorbitant ingrowth of fibrous tissues and thus negatively affect the patency. Currently, the optimal pore size is thought to be 60 μm for expanded PTFE vascular grafts, and 30 μm for PU grafts [22].

In our previous work [7], we have successfully prepared small-caliber (inner diameter of 4 mm) nanofibrous PU vascular grafts by electrospinning. We also found that these grafts offer three advantages: favorable fiber orientation and arrangement, excellent tensile properties, and high *in vitro* compatibility with endothelial cells.

In this study, we found that, as early as 4 weeks after the surgery, internal surfaces of the PU grafts were covered by endothelial cells. Although the cell layer on the PU graft was still irregular, this was still a substantial advancement compared with the PTFE grafts. Taken together, our findings suggest that endothelialization of the PU grafts is at all times superior to the PTFE grafts. The superior performance of the PU grafts may be attributed to their nanofibrous structures, which is similar to the host’s extracellular matrices (ECMs). Therefore, by effectively simulating the host ECMs, the PU graft scaffolds facilitate the surface precipitation of endothelial progenitor cells and their subsequent differentiation into mature endothelial cells, thereby preventing thrombogenesis and neointimal hyperplasia and eventually resulting into an increase in the patency rate.

It is generally accepted that proliferation of smooth muscle cells underlying the endothelium is primarily responsible for neointimal hyperplasia following vascular grafting. In our study, staining for α-SM actin indicated that, about 12 weeks after the surgery, the proliferation of smooth muscle cells inside the neointimas on the PU grafts became relatively stable; the neointimas did not further thicken between weeks 12 to 24, and the smooth muscle cells were orderly arranged with a high polarity orientation. By contrast, in the PTFE group the smooth muscle cells underlying the endothelium further thickened between weeks 12–24, and appeared disordered with a lower polarity orientation. These differences may be explained by the fact that the electrospun PU nano-fibers facilitate or induce the growth of smooth muscle cells along the directions of the fibers.

An optimal level of porosity is critical for the performance of vascular grafts. It is generally believed that optimal porosity of the internal surface can facilitate the anchoring of the neointima, while porosity of the external surface allows the tissue ingrowth leading to positional stability, prevention of graft distortion, facilitation of tissue ingrowth on the intimal surface, and promotion of neointimal formation [23]. In addition, pores also allow capillary vessels to form and penetrate the graft wall to accelerate the endothelialization of the internal surface [24].

On the other hand, excessive tissue ingrowth in the graft wall may lead to over-thickening of the neointima, or even severe stenosis or occlusion of the graft [24]. In our study, tissue extensively grew on the internal surfaces of the PTFE grafts, and neointimas gradually thickened suggesting an increase in tissue ingrowth on the walls. The lumens eventually became stenotic and even occluded. By comparison, at the early stage only a limited amount of tissue grew onto the walls of the PU grafts. This may be expected to delay early endothelialization, which, however, was not observed in this study. Similarly, at the late stage, only limited amount of tissue grew on the walls of PU grafts, and the neointimal thickness became constant, possibly limited by the connective tissues support and nutrition for proliferation of the smooth muscle cells, and thereby contributing to the relatively reduced neointimal hyperplasia and improved graft patency. It remains to be elucidated whether this comparative lack of tissue ingrowth onto the PU graft wall may cause other complications (e.g., inadequate mechanical strength on the long-term, formation of aneurysm / dissection, initiation of rupture). It is, indeed, important to find an optimal balance between the wall tissue ingrowth and long-term patency.

## Conclusions

In the present study, we confirmed that small-caliber nanofibrous PU vascular grafts exhibit good mechanical properties and *in vivo* biocompatibility, accelerate endothelialization process to prevent excessive neointimal hyperplasia, and lead to improved patency. These results indicate that these grafts may become promising vascular substitutes and warrant further studies and development.

## Abbreviations

CTA: Computed tomography angiography; ECMs: Extracellular matrices; PU: Polyurethane; PTFE: Polytetrafluoroethylene; PET: Polyethylene terephthalate; SEM: Scanning electron microscopy; vWF: von Willebrand factor.

## Competing interests

The authors declare that they have no competing interests.

## Authors’ contributions

ZH and SW conceived this study, participated in developing the study design and coordination, and helped to analyze the data and draft the manuscript. ZL participated in developing the study design, carried out the experiments, and analyzed the data. RL, YQ, LH, and WH performed the statistical analysis and helped to draft the manuscript. All authors read and approved the final manuscript.

## Pre-publication history

The pre-publication history for this paper can be accessed here:

http://www.biomedcentral.com/1471-2261/12/115/prepub
